# NMR Analyses and Statistical Modeling of Biobased Polymer Microstructures—A Selected Review

**DOI:** 10.3390/polym16050620

**Published:** 2024-02-24

**Authors:** Huai N. Cheng, Tetsuo Asakura, Koto Suganuma, Jose M. Lagaron, Beatriz Melendez-Rodriguez, Atanu Biswas

**Affiliations:** 1 USDA Agricultural Research Service, Southern Regional Research Center, New Orleans, LA 70124, USA; 2 Department of Biotechnology, Tokyo University of Agriculture and Technology, Koganei, Tokyo 184-8588, Japan; 3 Material Analysis Research Center, Teijin Ltd., Hino, Tokyo 191-8512, Japan; 4 Novel Materials and Nanotechnology Group, IATA, CSIC, Av. Agustín Escardino 7, 46980 Paterna, Valencia, Spain; 5 USDA Agricultural Research Service, National Center for Agricultural Utilization Research, Peoria, IL 61604, USA

**Keywords:** alginate, biobased polymers, chitosan, NMR, pectin, poly(lactic acid), poly(hydroxyalkanoate), sequence distribution, statistical modeling, tacticity

## Abstract

NMR analysis combined with statistical modeling offers a useful approach to investigate the microstructures of polymers. This article provides a selective review of the developments in both the NMR analysis of biobased polymers and the statistical models that can be used to characterize these materials. The information obtained from NMR and statistical models can provide insights into the microstructure and stereochemistry of appropriate biobased polymers and establish a systematic approach to their analysis. In suitable cases, the analysis can help optimize the synthetic procedures and facilitate the development of new or modified polymeric materials for various applications. Examples are given of the studies of poly(hydroxyalkanoates), poly(lactic acid), and selected polysaccharides, e.g., alginate, pectin, and chitosan. This article may serve as both a reference and a guide for future workers interested in the NMR sequence analysis of biobased materials.

## 1. Introduction

Biobased polymers, also known as agro-based or green polymers, are derived from renewable sources such as biomass, plants, and microorganisms. They can be chemically synthesized, modified from biological materials, or entirely biosynthesized by living organisms [1,2,3,4]. They offer several advantages over traditional petroleum-based polymers, including diminished environmental impacts, biodegradability, and a reduced reliance on fossil fuels. Examples of biobased polymers include cellulosic derivatives, starch-based polymers, seaweed polymers, proteins, poly(lactic acid), and poly(hydroxyalkanoates). Yet, for a full exploitation of the utility of biobased polymers, a detailed understanding of their compositions and microstructures is essential.

NMR can provide valuable insights into the structures of polymers at both the micromolecular and macromolecular levels. In particular, solution NMR can supply information on the chemical composition, homopolymer tacticity, copolymer sequences, end groups, branching, cross-linking, and (in selected cases) molecular weight of polymers [5,6,7,8,9,10,11,12]. However, some polymers may be complex and may require the concurrent use of additional techniques. One such technique entails statistical (or reaction probability) models [5,13,14]. These models can potentially rationalize the probabilities of different polymer configurations and sequences based on a number of factors, such as catalyst action, comonomer reactivity, and the reaction mechanism. Statistical models can also permit an understanding of polymerization processes and provide a theoretical framework to analyze NMR data in a logical way.

Among statistical models, Bernoullian and Markovian are the most well-known and are related to copolymerization equations [15,16], where Bernoullian, first-order Markovian, and second-order Markovian statistics correspond to random, terminal, and penultimate models of copolymerization, respectively. These models reflect polymerizations that are chain-end-controlled and are often used for NMR studies of synthetic polymers in the literature [5,7,9,10,13,14]. For example, the NMR sequence distributions of selected synthetic copolymers determined from NMR were found to agree with the first-order Markovian model and with reactivity ratios measured independently from copolymer composition data [17,18]. In addition to Bernoullian and Markovian models, the Coleman–Fox model [19,20,21] was formulated to explain the “block-like” behavior in the tacticity of some polymers. The enantiomorphic-site model [22,23] was designed for polymerizations that are catalytical-site-controlled, often used for homo- and copolymers made with Ziegler–Natta catalysts. A two-component model was devised for polypropylene tacticity [24]. Many other models used for synthetic polymers include Markovian with complex participation [25,26], reversible propagation [27,28,29], and bootstrap [30] models.

More integrated studies involving NMR analysis and statistical models were also reported, particularly those using more complex statistical models. The first combined use of a reaction probability model with NMR analysis was reported for the ethylene–propylene rubber system [31]. Since then, many other synthetic polymers have been studied in similar ways, including ethylene–propylene copolymers [32], propylene-1-butene copolymers [33], ethylene-1-butene copolymers [34], ethylene–vinyl chloride, ethylene–vinyl alcohol, ethylene–vinyl acetate copolymers [35], and other copolymers [36]. Many complex polymers may require mixture models [37] and perturbed models [38,39]. Through this approach, detailed information on homopolymer tacticity, copolymer sequences, sequence distribution, and (sometimes) polymerization mechanism can be obtained.

Because most biobased polymers tend to be complex, it is helpful to use combined NMR/statistical modeling to study their microstructures. The present article presents a review of the NMR/statistical modeling of selected biobased polymers. Different statistical models are needed for specific cases. In particular, NMR studies of poly(lactic acid), poly(hydroxyalkanoates), and applicable polysaccharides are covered in this work. Because of the compositional complexity of these biobased polymers, two-component and perturbed models are often needed for more precise analyses of NMR data.

## 2. Statistical Models

A summary of the many statistical models used for NMR analysis is given in Table 1. The simple models, including one-state Bernoullian (B), first-order Markovian (M1), second-order Markovian (M2), and enantiomorphic-site models (E), have been discussed in the preceding section and have also been reviewed in earlier publications [5,40,41,42].

The two-state models are used when two discrete states are involved in polymerization. The two states can be consecutive or concurrent [43]. The consecutive two-state model can be applied (for example) to a polymerization reaction where the catalytic site switches back and forth between two states as it polymerizes. The concurrent two-state model is similar to a mixture (two-component) model [37]. Many polymers are found to have multiple discrete polymeric components; a general methodology involving multistate models has been reported [37,44]. The analysis is facilitated when the NMR data of polymer fractions (e.g., from fractionation or chromatographic separation) are available.

Many industrial and biological polymers exhibit varying degrees of compositional heterogeneity. Compositional heterogeneity may also influence the polymer microstructure, such as tacticity, composition, and sequence distribution. Perturbed Bernoullian and Markovian models [38,39] have been designed as tools for the interpretation of NMR compositional, tacticity, and sequence data for heterogeneous polymers. These continuous models are especially useful when the composition covers a range of values, producing a chemical composition distribution. If the compositional heterogeneity is due to a mixture of specific polymer components, then the aforementioned mixture analysis can be performed as an alternative. Sometimes, the NMR data can be fitted to both perturbed and mixture models; in such cases, a goodness-of-fit criterion (e.g., the mean deviation between observed and fitted intensities) can be used. If available, other corroborative analytical techniques (such as fractionation, liquid chromatography, and size exclusion chromatography) may sometimes help confirm the nature of the heterogeneity.

Higher-copolymerization models [45] and kinetic models [46,47] represent a further level of complexity in polymer analysis. Thus far, they have tended to be used for synthetic polymers and not for biobased polymers.

**Figure 1:**
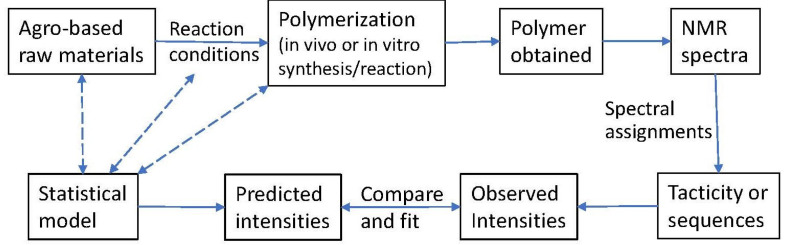
NMR analysis combined with statistical modeling.

The process whereby the combined NMR/statistical modeling approach is applied is summarized schematically in Figure 1. Biobased polymers can be made from agro-based raw materials through in vitro chemical synthesis {e.g., poly(lactic acid)}, natural growth and extraction {e.g., alginate, pectin, and chitin}, in vivo fermentation {e.g., poly(hydroxyalkanoates)}, or modification reactions {e.g., deacetylation of chitin to chitosan and modification of cellulose into cellulosic derivatives}. When a ^1^H or ^13^C NMR spectrum is obtained for a polymer, the spectral peaks need to be assigned to the proper polymer microstructure (e.g., homopolymer tacticity and copolymer sequences, like triad and tetrad sequences). The intensities are obtained via the integration of appropriate peaks in the NMR spectra. At the same time, a statistical model may be chosen based on the knowledge of the agro-based raw materials, the reaction conditions, and the nature of polymerization. Each statistical model is associated with a set of theoretical expressions for polymer sequences, leading to predicted values, which are fitted to the observed intensities via a simplex algorithm [32,36]. The goodness of fit between the observed and predicted intensities indicates how well suited the statistical model is to the polymer being studied. If the fit is poor, then a different (or improved) model should be attempted. If the fit is satisfactory, then the selected model provides a useful description of the polymer microstructure.

## 3. Poly(lactic acid) Tacticity

Poly(lactic acid) (PLA), an aliphatic polyester with thermoplastic properties, can be derived from renewable resources like corn, cassava, and sugarcane. As a well-known bioplastic, it shows favorable mechanical and environmental attributes, making it useful across diverse applications, including drug delivery systems, protein encapsulation, tissue engineering, sutures, and prostheses [48,49,50,51]. Integral to PLA’s physical and end-use properties is its stereochemistry. While racemic PLA exhibits a relatively low glass transition temperature (Tg), a mixture of poly(L-lactic acid) (PLLA) and poly(D-lactic acid) (PDLA) can form a stereo-complex, yielding an enhanced Tg and improved mechanical properties [51,52]. Consequently, the characterization of the stereochemistry of PLA is important as a means to understand the structure/property relationships in this family of materials.

As NMR is the preferred technique for studies of polymer stereochemistry and tacticity, many publications have appeared on PLA’s tacticity. These studies, conducted predominantly at the tetrad level for CH protons and carbon and, to some extent, at the hexad level for carbonyl carbon, have yielded valuable insights. The NMR peak assignments by Thakur et al. [53,54] are considered definitive and often cited; their papers [53,54] have also provided extensive reviews of previous publications in this area.

Quantum chemical calculations of chemical shifts [55,56] supported the prior PLA assignments. This approach constituted part of our studies that attempted to understand the origin of the tacticity splitting in the NMR spectra of PLA. In one paper [55], the ^1^H and ^13^C chemical shifts for dimer model compounds were calculated by averaging the occurrence probabilities obtained from the optimized conformational energies and the calculated chemical shift of each conformation. Good agreement between observed and calculated chemical shifts was obtained for the relative chemical shifts of isotactic and syndiotactic ^1^H and ^13^C NMR peaks of the dimer model compounds.

In the second paper [56], PLA dimer model compounds with different tacticities were synthesized and studied in detail by ^1^H and ^13^C NMR in three solvents, viz., CDCl_3_, CCl_4_, and d_6_-dimethyl sulfoxide. The complete assignments of peaks in the ^1^H and ^13^C NMR spectra were accomplished with the support of two-dimensional NMR techniques. Notably, the tacticity splitting of the dimer compounds exhibited minimal variance across the different solvents. For the elucidation of the origin of PLA tacticity splitting, the shifts were calculated and compared with the observed shifts by performing conformational energy calculations with quantum chemical methods and estimating the ^1^H and ^13^C chemical shifts for each conformation of the model compounds.

Another study [57] highlighted the impact of pyridine-d_5_ solvent on PLA’s NMR spectra due to the pyridine ring current effect and the electric field effect of the nitrogen lone pair. In order to achieve tacticity assignments, two-dimensional NMR spectra were obtained for poly(DL-lactic acid) (with a racemic ratio of L/D = 50/50), and relative peak intensities were compared across PLA samples. These studies enabled the enhanced resolution of methyl proton peaks and the partial assignment of these peaks [57].

In a recent work, a detailed NMR statistical modeling study of PLA tacticity was carried out [58] by preparing multiple PLA samples utilizing a tin catalyst, each possessing differing L,L-lactide (LL) and D,D-lactide (DD) ratios. When evaluating tetrad intensities with the pair-addition Bernoullian model, noticeable disparities between observed and calculated intensities were found. These disparities could be attributed to transesterification and racemization reactions inherent in the polymerization process. As a result, a new two-state model was formulated [58], encompassing both pair-addition Bernoullian and single-addition Bernoullian models (Table 2). This hybrid model produced a superior fit between observed and calculated data, offering a quantifiable assessment of the extent of transesterification and racemization in these samples.

**Figure 2:**
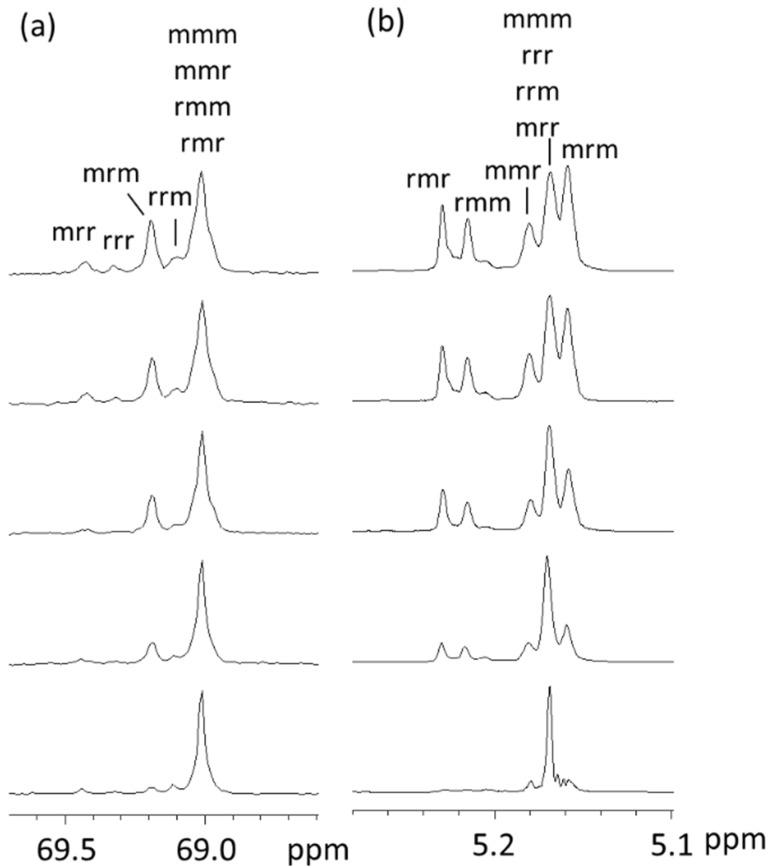
(**a**) ^13^C and (**b**) ^1^H NMR spectra of the CH groups of the PLA samples (from the top to the bottom: LL/DD = 50/50, 60/40, 70/30, 80/20, 90/10). (Reproduced from ref. [58]).

For example, the ^13^C and ^1^H NMR spectra of the CH groups of PLA samples made with LL/DD ratios of 50/50, 60/40, 70/30, 80/20, and 90/10 are shown in Figure 2, together with the assignments of the tacticity tetrads.

In Table 2, the observed intensities for the tetrads in the PLA 70/30 sample [58] were fitted to both the pair-addition Bernoullian model and the two-state model. As shown in Table 2, the one-state pair-addition Bernoullian model (model 1) gave a mean deviation of 1.2, but the two-state model (model 3) provided a notably better fit (mean deviation 0.4), indicating the preference for the two-state model.

## 4. Poly(hydroxyalkanoate) Comonomer Sequences

Polyhydroxyalkanoates (PHAs) are promising eco-friendly bioplastics because they are biodegradable and biocompatible and can be made either from natural materials [59,60,61] or chemically through the polymerization of hydroxyalkanoic acids or their derivatives [62,63]. PHA copolymers from bacterial sources exhibit properties that are contingent on their microstructures, which, in turn, can be tailored by utilizing distinct fermentation processes and feed materials. The most studied PHAs include poly(β-hydroxybutyrate) (PHB) and copolymers of 3-hydroxybutyrate (B) and 3-hydroxyvalerate (V). The insertion of comonomer V in PHBV has the advantage of reducing the stiffness, melting point, and crystallinity [59,61,64]. In recent years, PHAs with longer alkyl chains [65,66,67] and copolymers of B and higher alkyl hydroxyl esters [68,69] have also been reported.

Recently, a detailed study of the microstructure of PHBV was reported [70]. Previously, two ^13^C NMR peaks of hydroxyvalerate were assigned at the triad level. In the recent work, three ^13^C hydroxyvalerate peaks were resolved into triads; moreover, two ^13^C hydroxybutyrate peaks were resolved into four peaks. Through the use of eight copolymer samples spanning a broad composition range, all the resolved peaks were assigned to B-centered and V-centered triad sequences. These improved assignments permitted more precise values for both B-centered and V-centered triad sequence intensities to be determined and also enabled more accurate fitting of these values to statistical models.

**Figure 3:**
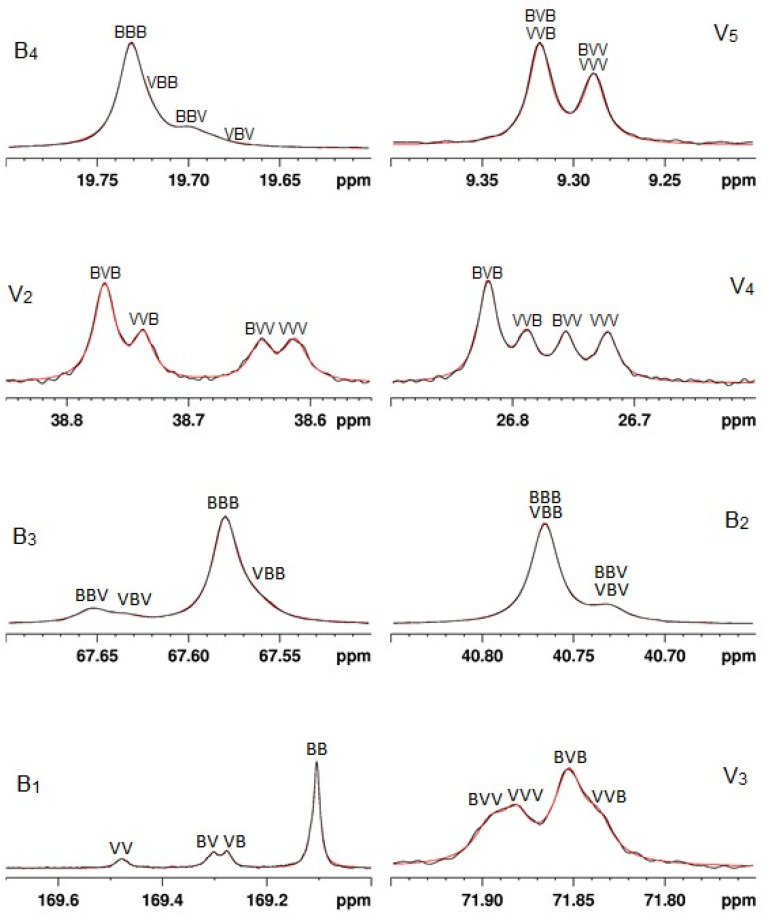
Expanded regions of the ^13^C NMR spectrum of PHBV sample 4 (V = 20%); black curves are the observed spectra, and red curves are the fitted spectra after curve deconvolution.

As an example, a sample of PHBV (sample 4) was obtained from fermented municipal wastewater as a feedstock. (It was one of eight PHBV samples derived from different feedstocks [70].) Once the microbial population reached a substantial level, the nutrient composition was changed to force the microorganisms to synthesize PHBV. The PHBV was then purified and isolated. The NMR spectra were obtained, and the triad sequences were derived through curve deconvolution of the NMR peaks (Figure 3). The observed triad intensities are shown in column 2 of Table 3.

The triad sequence intensities were analyzed using both the first-order Markovian (M1) model and the two-component Bernoullian (B/B) model (Table 3). The M1 model gave a somewhat large mean deviation (1.5), but the B/B model provided a much better fit (mean deviation 0.2). In view of the microbial feedstock, a mixture of PHBV polymers was probably generated, giving at least two separate polymer compositions, as suggested by the improved fit with the two-component B/B model.

## 5. Polysaccharide Sequence Determination

In recent years, polysaccharides have emerged as possible alternatives to synthetic plastics due to their abundant natural sources, renewability, and biodegradability [71,72]. Through appropriate modifications and/or processing techniques [73,74,75,76], polysaccharides can be transformed into a range of functional materials with properties suitable for packaging, biomedical applications, and various other industries.

As expected, the structure of polysaccharides can be studied with NMR [77,78,79], and the information available includes the composition, type, and degree of substituents, the presence of minor components and impurities, and sometimes even the number-average molecular weight. Moreover, NMR can be utilized in combination with other analytical techniques, such as methylation, esterification, fractionation, mass spectrometry, and chromatographic methods, to analyze complex polysaccharides or mixtures [80,81].

As for the use of NMR for the direct comonomer sequence determination of copolysaccharides, its feasibility depends on the polymers involved [77,78]. In some copolysaccharides, one or more NMR peaks in a saccharide residue of the polymer are split due to the sensitivity of the chemical shifts to the presence of different neighboring saccharide units. In this case, the split peaks need to be assigned to the appropriate sequences (e.g., diads, triads, or tetrads), and the percent distribution of the sequences can be determined by taking the areas of the resolvable peaks. However, if the NMR peaks of a copolysaccharide do not show resolvable peaks for different sequences, then NMR cannot be easily used for sequence determination for that copolymer. Three examples of polysaccharides where the NMR sequence peaks are resolvable are alginate, pectin, and chitosan. The NMR/statistical modeling of these polymers is shown below.

### 5.1. Alginate Mannuronic/Guluronic Sequence Analysis

Alginate is a naturally occurring polysaccharide found in various types of brown seaweed, including kelp and other marine brown algae [73,82,83]. It is used as a thickener, gelling agent, controlled release agent, coating, and stabilizing agent in the food industry, pharmaceuticals, and various other applications. Structurally, alginate is a linear copolymer made up of two types of monosaccharides: β-D-mannuronic acid (M) and α-L-guluronic acid (G). The sequence distribution of M and G residues along the polymer chain, as well as the overall composition (M-to-G ratio), determines many of the physical and chemical properties of alginate.

For alginate, ^1^H NMR can be used to compute overall composition (% M and % G), diad sequences, and G-centered triads, and ^13^C NMR can provide both M- and G-centered triad intensities. Previously, the NMR data of the whole polymer and fractions of alginate extracted from *Laminaria digitata*, as published in the literature [84,85], were analyzed [86], and four structural components were found: two mostly homopolymer blocks, one somewhat alternating copolymer block, and one or more random copolymer blocks.

An alternative approach is to use the hyphenated size exclusion chromatography (SEC)-NMR method [87,88]. The SEC instrument is connected to the NMR probe, and NMR spectra are obtained by stopping the flow during NMR data acquisition. Three commercial alginate samples were evaluated in this way. The NMR data were satisfactorily treated with two-component first-order Markov statistical models. The results were consistent with the earlier finding [86] that these alginate samples are compositionally heterogeneous, consisting of mixtures of components with different microstructures.

The NMR triad data for two commercial alginate samples with different M/G ratios were reported earlier by Kawarada et al. [89]. These data have been analyzed by both discrete and continuous models [90] and are reported in this work. As an example of the analysis, the triad data and model results for the sample with a high M/G ratio are shown in Table 4 (column 2). The one-component B model clearly did not fit the data well (column 3, with a mean deviation of 5.4). In contrast, both the discrete two-component B/B model (column 4) and the continuous perturbed B model (column 5) gave a mean deviation of 0.6, indicating a much better fit with the observed data. Thus, this alginate sample was heterogeneous in M/G sequence distribution, just like the earlier alginate samples from *L. digitata*.

### 5.2. Pectin Galacturonic Acid/Ester Sequence Analysis

Pectin is a well-known commercial product, typically produced from citrus peels and used as a gelling agent (especially in jams and jellies, dessert fillings, and sweets), as a food stabilizer in fruit juices and milk drinks, and as a source of dietary fiber [73,91,92]. It is commonly found in the cell walls of terrestrial plants. It has a complex structure, but the major functional unit is galacturonic acid. This acid can exist either as a carboxylic acid or as a methyl ester, and the plant can carry out this conversion enzymatically as needed. The gelling properties are related to the ratio of galacturonic acid to its ester. For high-methoxy (HM) pectin, pectin is usually mixed with sucrose to form a gel. For low-methoxy (LM) pectin, it is mixed with a calcium salt for gel formation. Thus, the amount and the placement of the acid and the ester along the polymer chain are important information for product development and formulation.

NMR is a good technique to measure the amount of acid/ester present in pectin, as well as the heterogeneity of their placements along the polymer chains [77]. It is known that selected peaks in ^1^H and ^13^C spectra are split by the acid/ester sequence effects so that the triad sequence distributions can be obtained. Analyses were previously reported for selected pectin samples using statistical modeling [93,94], and the data were shown to fit well to both discrete and continuous models. For illustration, the reported triad distribution data for an HM pectin sample [94] extracted from lemon peel are shown in column 2 of Table 5, where G and E denote galacturonic acid and the ester, respectively. The data were re-analyzed in this work for three types of Bernoullian (B) models: a simple B model, a two-component (B/B) model, and a continuous perturbed B model. From the analysis shown in Table 5, the simple B model (column 3) gave a mean deviation of 1.2, the two-component B/B model (column 4) 0.6, and the perturbed B model (column 5) 0.4. Thus, the NMR triad sequence data suggest that the particular HM pectin sample was compositionally heterogeneous, and the NMR data could be fitted with either the two-component B/B model or the perturbed B model.

A second example can be given using an LM pectin sample. The reported triad distribution data for such a sample (also from lemon peel) [94] are shown in column 2 of Table 6, and the analysis results of these data using the same three models are given in columns 3–5 of Table 6. In this case, the mean deviations for the three models (B, B/B, and perturbed B) were 0.8, 0.1, and 0.1, respectively, also suggesting that the NMR triad sequence data conformed better to the two-component B/B model or the continuous perturbed B model.

The above analyses suggest that both HM pectin and LM pectin samples extracted from lemon peels are compositionally heterogeneous. In a separate work [87], pectin fractionation from the same source material was analyzed in combination with NMR, and this is another way to confirm the compositional heterogeneity. Thus, NMR and statistical modeling can be helpful for the analysis of citrus pectin samples.

### 5.3. Sequence Analysis of Partially Deacetylated Chitosan

Chitin is abundant in the exoskeletons of crustaceans, insects, and fungi. Structurally, it is a homopolymer of 2-acetamido-2-deoxy-β-D-glucopyranose (GlcNAc). Chitosan is obtained by partially deacetylating chitin and may be regarded as a copolymer of GlcNAc units and 2-amino-2-deoxy-β-D-glucopyranose (GlcN) units [73,95,96]. Chitosan is a versatile polymer with a myriad of applications. Its unique properties, including biocompatibility, biodegradability, and non-toxicity, make it invaluable in various applications. It also has notable antimicrobial properties, making it a possible additive for use in wound healing, tissue engineering, and other medical products. Moreover, it is employed in the food and pharmaceutical industries for its ability to function as a natural preservative and a controlled delivery vehicle.

For partially deacetylated chitosan, the NMR spectra can detect chemical shift differences for the different sequences of acetylated and deacetylated units. Previously, the ^1^H and ^13^C NMR spectra of chitosan were published by Varum et al., who also reported the triad sequence intensities of selected samples [97,98]. The triad sequence intensities for one sample are shown in column 2 of Table 7. In an earlier analysis [99], the NMR data of chitosan were shown to be compatible with a compositionally heterogeneous polymer. In this work, the data were re-analyzed with three Bernoullian models. The analysis is given in Table 7, where A = acetylated unit (GlcNAc residue) and D = deacetylated unit (GlcN residue).

From the results in Table 7, the use of the simple B model clearly gave a rather large mean deviation of 3.0 (column 3). In the two-component B/B model (column 4), a second minor component (9.4%) consisting of mostly deacetylated units (P_A_ = 0.009) was incorporated, and the mean deviation was reduced to 1.5. The best result was found for the perturbed B model (column 5), where the mean deviation was cut down to 0.4. Thus, this chitosan sample was heterogeneous in composition, as shown by the analysis of NMR data with statistical modeling.

### 5.4. Comments

It may be noted that only some biobased polymers are amenable to being studied by the NMR/statistical methods described herein. First, the polymer in question should have a structure that contains only two or three repeating units, thereby generating a manageable number of structural sequences. (Thus, a protein that is composed of 20 amino acids may be too complex to be studied by NMR/statistical model approaches.) Secondly, the different structural sequences should give detectable differences in the NMR spectra, typically through chemical shift differences. If the shift difference in a polymer spectrum is too small to be resolved by NMR, then NMR microstructural studies of this polymer will be difficult. However, as the NMR instrumentation continues to improve in sensitivity, resolution, and magnetic field strength [100,101], it is possible that small chemical shift differences may be resolvable in the future, thereby rendering more polymer microstructures accessible to NMR sequence analysis.

## 6. Conclusions

Because many biobased polymers are derived from agro-based materials, they may contain complex structures or a mixture of related polymers. A simple NMR analysis of these polymers may not give the full picture of the polymeric structure. Through the combination of NMR analysis and statistical modeling, it may be possible to gain a better understanding of the detailed polymer composition and microstructure (such as compositional heterogeneity, sample variability, and presence of mixtures). In this review, the NMR/statistical modeling of several biobased materials has been summarized in order to show how detailed structural information can be derived from the NMR data. Thus, the information given in this review may be helpful to future workers who study biobased polymers with NMR.

Another consideration in the use of NMR for polymer analysis is the relatively high expense of NMR instrumentation. Thus, when NMR data are obtained from biobased materials, it is beneficial to extract the maximum amount of information available from the data. This is one of the benefits of using statistical modeling. For example, the NMR information obtained on copolymer sequence distribution, compositional heterogeneity, and sample variability may be helpful in the process improvement of the extraction and purification of these polymers and in the structure/property correlation studies of the end products.

## Figures and Tables

**Table 1:** A summary of different statistical models that can be used for NMR analysis. The letters B, M1, M2, and E refer to Bernoullian, 1st-order Markovian, 2nd-order Markovian, and enantiomorphic-site models, respectively.

NMR Information	Statistical Models	References
Homopolymer tacticity and copolymer sequence	1. One-component models (discrete) a. Chain-end control: B, M1, M2 b. Catalytic-site control: E model c. Both end and site control: EM1, EM2	[5,40,41,42]
	2. Two-component models (discrete) a. Consecutive B/B, B/E, E/E b. Concurrent B/B, B/E, E/E	[43]
	3. Multi-component models (discrete) a. Consecutive multisite models b. Concurrent multisite models	[37,44]
	4. Perturbed models (continuous) a. Symmetric B and M1 models b. Non-symmetric B and M1 models c. General-case models	[38,39]
Terpolymers and Tetrapolymers	Higher-copolymerization models	[45]
Branched polymers and more complex polymers	Kinetic models	[46,47]

**Table 2:** Theoretical expressions for tetrad fractions in the various statistical models used for the NMR analysis of the polymerization of mixtures of L,L- and D,D-lactides, together with observed and calculated intensities for a sample of PLA (70/30) (adapted from ref. [58]).

Tetrad	Model 1 ^a^	Model 2 ^b^	Model 3 ^c^	Obsd. %	Calc. % Mod. 1	Calc. % Mod. 3
mmm	(p_2_^2^ + q_2_^2^ + p_2_^3^ + q_2_^3^)/2	p_1_^4^ + q_1_^4^	f_2_[(p_2_^2^ + q_2_^2^ + p_2_^3^ + q_2_^3^)/2] + f_1_[p_1_^4^ + q_1_^4^]	39.9	40.1	39.9
mrm	p_2_q_2_	2p_1_^2^q_1_^2^	f_2_ p_2_q_2_ + f_1_ [2p_1_^2^q_1_^2^]	21.4	24.0	21.3
mmr	p_2_q_2_/2	p_1_^3^q_1_ + p_1_q_1_^3^	f_2_ p_2_q_2_/2 + f_1_ [p_1_^3^q_1_ + p_1_q_1_^3^]	11.3	12.0	11.6
rmm	p_2_q_2_/2	p_1_^3^q_1_ + p_1_q_1_^3^	f_2_ p_2_q_2_/2 + f_1_ [p_1_^3^q_1_ + p_1_q_1_^3^]	10.9	12.0	11.6
rmr	p_2_q_2_/2	2p_1_^2^q_1_^2^	f_2_ p_2_q_2_/2 + f_1_ [2p_1_^2^q_1_^2^]	12.0	12.0	11.3
rrm	0	p_1_^3^q_1_ + p_1_q_1_^3^	f_1_ [p_1_^3^q_1_ + p_1_q_1_^3^]	2.5	0	1.5
mrr	0	p_1_^3^q_1_ + p_1_q_1_^3^	f_1_ [p_1_^3^q_1_ + p_1_q_1_^3^]	1.5	0	1.5
rrr	0	2p_1_^2^q_1_^2^	f_1_ [2p_1_^2^q_1_^2^]	0.7	0	1.2
				MD	1.2	0.4
				Reaction probabilities	p_2_ = 0.6	p_1_ = 0.66 f_1_ = 0.12 p_2_ = 0.65 f_2_ = 0.88

^a^ Model 1 = pair-addition Bernoullian model; reaction probabilities p_2_ = LL/(LL + DD) and q_2_ = DD/(LL + DD). ^b^ Model 2 = single-addition Bernoullian model; reaction probabilities p_1_ = L/(L + D) and q_1_ = D/(L + D). ^c^ Model 3 = two-state (pair-addition + single-addition Bernoullian models); the fractions of single addition and pair addition are f_1_ and f_2_, respectively, where f_1_ + f_2_ = 1.

**Table 3:** Observed and calculated triad intensities (in mole %) of PHBV sample 4 as fitted by first-order Markovian (M1) and two-component (Bernoullian/Bernoullian) mixture models, adapted from ref. [70].

Triad	Obsd %	M1 Model Expressions *	Calc. %	Two-Component B/B Model Expressions **	Calc. %
VVV	4.7	kP_BV_P_VV_^2^	4.7	w_1_P_V1_^3^ + w_2_P_V2_^3^	4.7
BVV + VVB	8.3	2kP_BV_P_VV_P_VB_	11.6	2w_1_P_V1_^2^(1 − Pv_1_) + 2w_2_P_V2_^2^(1 − Pv_2_)	8.3
BVB	9.7	kP_VB_^2^P_BV_	7.1	w_1_P_V1_(1 − Pv_1_)^2^ + w_2_P_V2_(1 − Pv_2_)^2^	9.9
VBV	3.9	kP_BV_^2^P_VB_	2.2	w_1_P_V1_^2^(1 − Pv_1_) + w_2_P_V2_^2^(1 − Pv_2_)	4.1
BBV + VBB	20.3	2kP_VB_P_BB_P_BV_	21.4	2w_1_P_V1_(1 − Pv_1_)^2^ + 2w_2_P_V2_(1 − Pv_2_)^2^	19.9
BBB	53.1	kP_VB_P_BB_^2^	53.1	w_1_(1 − P_V1_)^3^ + w_2_(1 − P_V2_)^3^	53.1
		mean deviation	1.5	mean deviation	0.2
		P_BV_	0.168	P_V1_	0.134
		P_VB_	0.551	w_1_	0.802
		r_1_ r_2_	4.03	P_V2_	0.610
				w_2_	0.198

* M1 reaction probabilities are P_BV_ and P_VB_, where k = (P_BV_ + P_VB_)^−1^; ** Bernoullian reaction probabilities are P_V1_ and P_V2_ for V insertion in components 1 and 2, respectively. For B insertion, the Bernoullian reaction probabilities are (1 − P_V1_) and (1 − P_V2_), respectively. Note that w_1_ is the fraction of component 1, and the fraction of component 2 (w_2_) is equal to 1 − w_1_.

**Table 4:** The triad sequence intensities (in mole %) of a commercial alginate sample; observed triad data were taken from ref. [89], and model calculations were performed for this work.

NMR Triad	Obsd %	Discrete Models		Continuous Model
		Calc % (for B)	Calc % (for B/B)	Calc % (for Perturbed B)
MMM	39	39	39	39
MMG	17	29	19	18
GMG	8	5	7	7
MGM	10	14	9	9
GGM	14	11	14	15
GGG	12	2	12	12
Mean dev.		5.4	0.6	0.6
Reaction probabilities		P_M_ = 0.731	Component 1: w_1_ = 0.592 P_M_ = 0.858 Component 2: w_2_ = 0.408 P_M_ = 0.338	P_M_ = 0.648 σ = 0.253 τ = −0.004

**Table 5:** The triad sequence analysis of an HM pectin sample; observed triad data were taken from ref. [94], and model calculations were performed for this work.

NMR Triad	Obsd. %	Discrete Models		Continuous Model
		Calc % (for B)	Calc % (for B/B)	Calc % (for Perturbed B)
EEE	32.4	32.4	32.5	32.4
EEG	28.0	29.5	28.0	27.5
GEG	6.4	6.7	7.1	6.7
EGE	13.0	14.8	14.0	13.8
GGE	14.0	13.5	14.0	13.4
GGG	6.2	3.1	4.4	6.2
Mean dev.		1.2	0.6	0.4
Reaction probabilities		P_E_ = 0.687	Component 1: w_1_ = 0.793 P_E_ = 0.724 Component 2: w_2_ = 0.207 P_E_ = 0.489	P_E_ = 0.675 σ = 0.118 τ = −0.008

**Table 6:** The triad sequence analysis of an LM pectin sample; observed triad data were taken from ref. [94], and model calculations were performed for this work.

NMR Triad	Obsd. %	Discrete Models		Continuous Model
		Calc % (for B)	Calc % (for B/B)	Calc % (for Perturbed B)
EEE	2.1	1.6	2.4	2.5
EEG	10.6	9.6	10.6	10.6
GEG	13.4	14.1	13.3	13.3
EGE	5.5	4.8	5.3	5.3
GGE	26.7	28.2	26.7	26.6
GGG	41.7	41.7	41.7	41.7
Mean dev.		0.8	0.1	0.1
Reaction probabilities		P_E_ = 0.253	Component 1: w_1_ = 0.337 P_E_ = 0.586 Component 2: w_2_ = 0.159 P_E_ = 0.414	P_E_ = 0.264 σ = 0.0928 τ = 0.0003

**Table 7:** The triad sequence analysis of partially deacetylated chitosan; observed triad data were taken from refs. [97,98], and model calculations were performed for this work.

NMR Triad	Obsd. %	Discrete Models		Continuous Model
		Calc % (for B)	Calc % (for B/B)	Calc % (for Perturbed B)
AAA	15	15	16	15
AAD	28	27	25	28
DAD	10	12	10	9
ADA	14	13	13	14
DDA	16	23	20	17
DDD	17	10	17	17
Mean dev.		3.0	1.5	0.4
Reaction probabilities		P_A_ = 0.531	Component 1: w_1_ = 0.906 P_A_ = 0.357 Component 2: w_2_ = 0.094 P_A_ = 0.009	P_A_ = 0.547 σ = 0.083 τ = −0.031

## Data Availability

The data presented in this study are available on request from the corresponding author.
